# Slight Pro-Inflammatory Immunomodulation Properties of Dendritic Cells by* Gardnerella vaginalis*: The “Invisible Man” of Bacterial Vaginosis?

**DOI:** 10.1155/2016/9747480

**Published:** 2016-02-17

**Authors:** Thomas Bertran, Patrick Brachet, Marjolaine Vareille-Delarbre, Julie Falenta, Annie Dosgilbert, Marie-Paule Vasson, Christiane Forestier, Arlette Tridon, Bertrand Evrard

**Affiliations:** ^1^UFR Médecine-Pharmacie, Clermont Université, Université d'Auvergne, UMR 1019 UNH, ECREIN, Laboratoire d'Immunologie, 63001 Clermont-Ferrand, France; ^2^INRA de Theix, UMR 1019 UNH, ECREIN, 63122 Saint-Genès-Champanelle, France; ^3^Service d'Immunologie, CHU Clermont-Ferrand, 63001 Clermont-Ferrand, France; ^4^UFR Pharmacie, Clermont Université, Université d'Auvergne, UMR 1019 UNH, ECREIN, Laboratoire de Biochimie, 63001 Clermont-Ferrand, France; ^5^UFR Pharmacie, Clermont Université, Université d'Auvergne, UMR CNRS 6023 LMGE, Laboratoire de Bactériologie, 63001 Clermont-Ferrand, France

## Abstract

Bacterial vaginosis (BV), the most common genital infection in reproductive-aged women, is associated with increased risk of sexually transmitted infections. Its etiology remains unclear, especially the role of* Gardnerella *(*G.*)* vaginalis*, an anaerobic bacterium characteristic of the BV-alteration of the vaginal ecosystem. In the genital mucosa, dendritic cells (DCs) sense bacteria of the microenvironment* via* receptors and then orchestrate the immune response by induction of different T cell subtypes. We investigated the interactions between* G. vaginalis *and human monocyte-derived DCs using a wide range of bacterial concentrations (multiplicity of infection from 0.01 to 100), and the effects of this pathogen on PHA-induced lymphocyte proliferation. As observed by electron microscopy and cytometry,* G. vaginalis* reduced the internalization ability of DCs by forming extracellular clusters and induced neither DC maturation, nor DC secretion of cytokines, except at the highest dose with a very early DC maturation state. The same profile was observed on lymphocytes with significant increases of proliferation and cytokine secretion only at the highest bacterial concentration. Our findings indicate that* G. vaginalis *possesses slight immune-stimulating activities against DCs and T cells, reflecting thus a defective inflammatory response and giving rise to the atypical, non- or low-grade, inflammatory clinical disease profile.

## 1. Introduction

Bacterial vaginosis (BV) is the most common low genital infection among reproductive-aged women, with a prevalence of 29% among 14- to 49-year-old US women and almost 40% in individuals at high risk for sexually transmitted infections (STIs) [1]. BV is associated with serious medical complications, including adverse pregnancy outcomes, endometritis, and pelvic inflammatory disease such as endometriosis [2, 3]. BV also increases women's risk of acquiring STIs, particularly HIV infections [4, 5].

Clinically, one-half of BV-positive women are asymptomatic while the others suffer only from mild symptoms, such as homogeneous white vaginal discharge and amine (fishy) odor [6]. These signs are associated with a vaginal pH > 4.5 and the presence of characteristic “clue cells” on microscopic examination. These four manifestations constitute Amsel's clinical criteria [6]. The microbiological diagnosis of BV is usually based on Nugent's score, which includes assessment of lactobacilli by Gram's stain of vaginal fluid samples. BV is associated with an alteration of the vaginal ecosystem, characterized by a decrease in hydrogen peroxide-producing* Lactobacillus (L.) *species such as* L. crispatus *and* L. jensenii* and a concomitant increase in polymicrobial anaerobic bacteria like* Gardnerella* (*G*.)* vaginalis *[7, 8].

The microbial etiology of BV is unclear and a matter of debate [9]. Two opposing hypotheses exist [10]. In the monomicrobial hypothesis, historically the first one,* G. vaginalis* is the single, specific etiologic agent of BV [11]. In the polymicrobial hypothesis, which has gained general acceptance in the last 20 years,* G. vaginalis* acts synergically with other anaerobes to unbalance the vaginal flora and trigger the disease [12]. Vaginal inoculation experiments in the monkey show thus that the co-occurrence of anaerobes and* G. vaginalis *is required to induce BV [13]. Moreover,* G. vaginalis* is frequently isolated in healthy women without BV [14]. Nevertheless, recent works have relaunched the debate by confirming its importance in the pathophysiology of the disease.* G. vaginalis* predominates in vaginal BV-associated biofilms, which are implicated in persistent BV, thus constituting a major factor of resistance to standard treatment [15].

Dendritic cells (DCs) are professional antigen presenting cells (APCs) which, by inducing both tolerance and immunity, are critical for the orchestration of the adaptive immune response [16]. Immature DCs reside in peripheral mucosa, where they sense the microenvironment* via* pattern recognition receptors (PRRs) that recognize pathogen-associated molecular patterns (PAMPs). PRRs include toll-like receptors (TLRs) and C-type lectin receptors (CLRs) [17]. PRR stimulation triggers a DC maturation process with up-regulation or down-regulation of membrane molecules (CD83, CD86 and HLA-DR, and DC-SIGN and Mannose Receptor, resp.) and cytokine production. DC activation by several PAMPs, via distinct PRRs with antagonistic or synergistic effects, modulates their differentiation, which secondarily determines the polarization of the effector T cell responses,* that is,* the balance between Th1, Th2, Th17, and T regulatory (Treg) subsets [18]. Cytokine production by DCs is an important factor in this process. IL-12 production drives polarization towards Th1 cells, whereas synthesis of IL-1*β*, IL-6, TGF*β* and IL-23, and IL-10 promotes induction of Th17 or Treg cells, respectively [19]. These different stages of the immune response have been recently described in the human genital mucosa. Notably, in both the upper and lower tracts, several DC subsets exist and express specific TLRs, such as TLR-6, TLR-7, and TLR-8, and CLRs, such as langerin and DC-SIGN, and are able to induce different T cell subpopulations [20–22].

The effects of mucosal fluids from women with BV or healthy flora, without analysis of implicated bacterium species, were examined on DC function [23, 24]. BV samples induced IL-12 and IL-23 production, as well as expression of maturation markers (HLA-DR, CD40, and CD83) by monocyte-derived DCs (moDCs). Concerning T cells, there has been yet no investigation on the impact of BV on the polarization of the different lymphocyte subpopulations. Only one study reported effects of BV on the percentage of Treg cells in peripheral blood mononuclear cells (PBMCs). However, this study did not test the possibility of a specific impact of* G. vaginalis* and it did not objectify differences in the distribution of Treg in BV+* versus* BV− HIV-negative women, decreased Treg being solely observed in BV+/HIV+ women compared to BV−/HIV+ women [25]. Many studies have attempted to measure cervicovaginal production of cytokines in BV, but disparate results were obtained. Most articles reported an elevation in IL-1*β*, and less consistently in IL-6 and IL-8, in BV-affected women [26–29]. Additionally, BV mucosal fluid was found to increase proliferation of T cells in allogeneic mixed-leukocyte reaction (MLR) [23]. Finally, the specific effects of* G. vaginalis* on DC and T cells have never been evaluated yet.

Unlike conventional vaginitis that is characterized by burning, dysuria, dyspareunia, and frequent pruritus, BV causes scant inflammatory signs without primary pain or pruritus in affected women [14]. Likewise, a relative paucity of inflammatory cells and a near normal number of vaginal neutrophils are characteristic of BV status. In view of the literature data, we hypothesized that BV corresponds to a unique local immunological environment, with a low-grade inflammation, potentially mediated by so far unknown immunomodulatory mechanisms of action of* G. vaginalis* on the vaginal immune system, particularly on the DCs and T cells. In the present study, we investigated this hypothesis in* in vitro *models by monitoring (i) the internalization, maturation, and cytokine secretion of moDCs; (ii) lymphocyte proliferation and subset cytokine production, after cell exposure to* G. vaginalis* or to commensal or pathogenic microorganisms potentially found in the vaginal mucosa.

## 2. Material and Methods

### 2.1. Bacterial Strains and Culture Conditions

*G. vaginalis *ATCC14018 was grown in brain heart infusion (Biomérieux) supplemented with maltose (0.1%), glucose (0.1%), yeast extract (1%), and horse serum (10%) in 5% CO_2_ at 37°C for 72 h.* L. reuteri* ATCC23272 was grown in De Man, Rogosa, Sharpe (MRS) medium (BD Difco*™*) at 37°C overnight.* Candida albicans *ATCC10231 was grown in Sabouraud broth at 37°C overnight. Microbial cells were harvested by centrifugation (11,000 ×g for 10 min), and the pellet was washed twice and then resuspended in RPMI 1640 (Cambrex Bio Science). Optical density (OD) measurements were performed at 620 nm to adjust the final concentration of the microbial suspension, and the exact number of colony forming units (CFU) was determined by plating serial dilutions of the inocula onto adapted agar plates (Columbia 5% Sheep Blood Agar, MRS, or Sabouraud). Before being added to the cell samples, the microbial cells were inactivated by exposure to UV for 1 h. The effectiveness of the inactivation was evaluated by plating 20 *μ*L of the irradiated inocula on adapted agar plates.

### 2.2. Ethics Statement

The human cells used in this study were generated from the buffy-coat of healthy volunteers obtained from the local French blood agency (Etablissement Français du Sang, EFS, Saint-Etienne). It is a statutory requirement that blood donors be given full necessary information (article R.1221-5 of the Public Health Code, 12/01/2009 and 11/06/2006 decrees). Written informed consent was obtained by the EFS from all volunteers involved in our study.

### 2.3. *In Vitro* Differentiation of Monocyte-Derived Dendritic Cells

DCs were generated from PBMCs. Briefly, PBMCs were isolated from the buffy-coats of healthy volunteers by Ficoll-Histopaque (Sigma) density gradient centrifugation. PBMCs were washed twice in RPMI 1640 and resuspended at a final concentration of 5 × 10^7^ cells per mL of phosphate buffered saline (PBS) supplemented with 2% fetal calf serum (FCS, Biowest-Abcys) and 1 mM EDTA. Monocytes were purified by negative selection using the EasySep^®^ Human Monocyte Enrichment Kit, as recommended by the manufacturer (StemCell Technologies). They were then cultured for 5 days in RPMI 1640 supplemented with 1% L-glutamine (Sigma), 10% FCS, and 0.5% penicillin-streptomycin (Sigma), in the presence of 500 U/mL IL4 (R&D systems) and 800 U/mL granulocyte-macrophage colony-stimulating factor (GM-CSF, R&D systems). After 3 days of incubation, one-half volume of fresh culture medium containing 2x concentrations of IL4 and GM-CSF was added to each well.

### 2.4. Electron Microscope Observations

DCs obtained as previously described were plated in a sterile 12-well plate at a concentration of 1 × 10^6^ cells/mL.* G. vaginalis *was added to wells at a multiplicity of infection (MOI) of 10 for 1 h (Scanning Electron Microscopy, SEM) and at a MOI of 0.01, 1, or 100 for 3 h (Transmission Electron Microscopy, TEM). Cells were harvested, centrifuged (400 ×g for 10 min), rinsed with Natrium Cacodylate (0.2 M pH 7.4) for 10 min, and then fixed at 4°C overnight with glutaraldehyde 1.6% in Natrium Cacodylate buffer. The samples were then rinsed, postfixed with 1% Osmium Tetroxide (1 h, room temperature), rinsed again, and dehydrated with graded series of ethanol (70 to 100%) and eventually with 100% hexamethyldisilazane. Finally, after overnight drying, samples were placed on a Jeol SEM filter and metallised with carbon (40 s). For TEM, dried samples were embedded in a polymerized 2 mm thick Epon coating, and ultrathin sections were picked up with Formvar-coated copper grids (300 mesh). Sections were counterstained with 4% aqueous uranyl acetate. For negative staining, bacteria were grown overnight in M63B1-0.4% Glu medium and negatively stained with 2% phosphotungstic acid on Formvar-coated copper grids (300 mesh). Images were captured at the Centre d'Imagerie Cellulaire Santé (CICS) of the Université d'Auvergne with a Jeol JSM-6060LV (SEM) and a Hitachi H-7650 (TEM).

### 2.5. Flow Cytometry Analysis of DC Maturation and Viability

On day 6, the immature DCs from each well were harvested, pooled, centrifuged, and reseeded at 1 × 10^5^ cells/mL. UV-killed bacteria were then added at 10 *μ*L of suspension per well to reach a final concentration ranging from 10^3^ to 10^7^ CFU/mL,* that is,* a MOI between 0.01 and 100. Lipopolysaccharide (LPS) from* Escherichia coli* (Sigma) at a final concentration of 100 ng/mL was used as positive control. Immature DCs without addition of LPS or bacteria were used as negative control. After 48 h of maturation at 37°C in a 5% CO_2_ atmosphere, DCs were collected, centrifuged, and resuspended in PBS with 1% bovine serum albumin (BSA, Sigma). Cell surfaces were stained with the appropriate fluorescence-labeled murine antibodies: APC-Cy7-conjugated anti-CD14 (LPS coreceptor specific to monocytes), PE-conjugated anti-CD86 (costimulatory molecule, activation marker), V450-conjugated anti-HLA-DR, PerCP-Cy5.5-conjugated anti-DC-SIGN (DC-specific ICAM-3 grabbing-nonintegrin or CD209, member of the CLR family, specific marker of immature DCs), Alexa Fluor^®^ 488-conjugated anti-MR (Mannose Receptor or CD206, member of the CLR family, specific marker of immature DCs and macrophages), and streptavidin APC-conjugated anti-TLR4 (biotin antibody, an LPS receptor with activation functions). Antibodies were obtained from BD Biosciences, except anti-MR (Biolegend). Corresponding murine isotype-matched and non-labeled antibodies (BD Biosciences or Biolegend) were used as controls. The cells were analyzed by a BD-LSRII flow cytometer with FACSDiva Software (BD Biosciences) at the CICS. Fluorescence compensation adjustments were performed. Gates were set on living DCs based on their forward/side scatter (FSC/SSC) properties. The analysis was halted at a count of 3,000 DCs. The level of staining was expressed as the mean fluorescence intensity (MFI). Culture supernatants were collected and stored at −20°C until cytokine analysis. To determine cell viability, dye LIVE/DEAD^®^ beads (Fixable Blue Dead Cell Stain Kit, for UV excitation Life technologies) were added to cells and used as markers of dead cells. DC mortality was assessed by gating together dead cells and live DCs on SSC/FSC diagram. Two other gates were created from this gate to separate the two populations. From the dead cell gate, cells that expressed LIVE/DEAD marker were considered as effectively dead. This dead cell number was expressed as a ratio of the first created overall gate to obtain a dead DC percentage.

### 2.6. Lymphocyte Proliferation Assays

The mitogenic response to plant lectins, as phytohemagglutinin A (PHA), is conventionally used to measure cell-mediated immunity in mammals in general and especially in humans [30]. These tests are named lymphocyte proliferation assays or lymphocyte transformation test (LTT). They were performed here to study the functional properties of* G. vaginalis *and especially its capabilities to modulate PHA-induced T cell proliferation. On day 1, PBMCs collected from a buffy-coat as previously described were adjusted to a concentration of 1 × 10^6^ cells per mL of complete medium,* that is,* RPMI 1640 supplemented with 1% L-glutamine and 10% FCS. The cell suspension was deposited in a sterile 96-well plate (100 *μ*L per well). Each measurement was done in triplicate. Polyclonal proliferation of lymphocytes was induced with 2 *μ*g/mL of PHA (Sigma). Cells without PHA or bacteria were used as a negative control and cells with PHA without bacteria as a positive one.* G. vaginalis *was added to other PHA-treated wells to obtain concentrations ranging from 10^3^ to 10^7^ CFU/mL. After a 72 h incubation at 37°C under 5% CO_2_, 1 *μ*Ci of tritiated thymidine (Perkin Elmer) was added to each well and the cells were further incubated for 4 h. Labeling was stopped by cooling the plate to 4°C. The cells were then collected under vacuum onto a Whatman filter paper and incorporation of tritiated thymidine was measured using a *β* counter (Tri-Carb 2300TR, Canberra-Packard). Proliferation results were expressed as mean cpm (counts per min) values of triplicate measurements. Identical incubations, however, without addition of tritiated thymidine, were carried out in parallel to collect supernatants for cytokine quantification.

### 2.7. Cytokine Quantification

For DC maturation experiments, the cytokines IL-10, TNF-*α*, IFN-*γ*, and IL-12p70 were assayed in culture supernatants with Biolegend enzyme-linked immunosorbent assay (ELISA) kits according to the manufacturer's instructions. For lymphocyte proliferation assays, the cytokines IFN-*γ*, IL-4, IL-17A, IL-10, IL-12p70, and TNF-*α* were quantified in supernatants of PBMC cultures using Pro Human Cytokine Group 1 6-Plex 1 × 96 kit (Bio-Rad) on a Bio Plex^®^ 200 (Bio-Rad).

### 2.8. Statistical Analysis

All data were expressed as means + SD. After variance dispersion test, three different statistical tests were performed. Two-way ANOVA with* post hoc* Bonferroni test, Friedman's test with Nemenyi's group, and Kruskal-Wallis test with Dunn test were used to analyze the significant effect of bacteria on DCs or PBMCs with XLStat 7.5.2 software (Addinsoft, Paris, France). *p* values lower than 0.05 were considered statistically significant.

## 3. Results

### 3.1. Electron Microscopy Observation of* G. vaginalis*-DC Interaction

**Figure 1:**
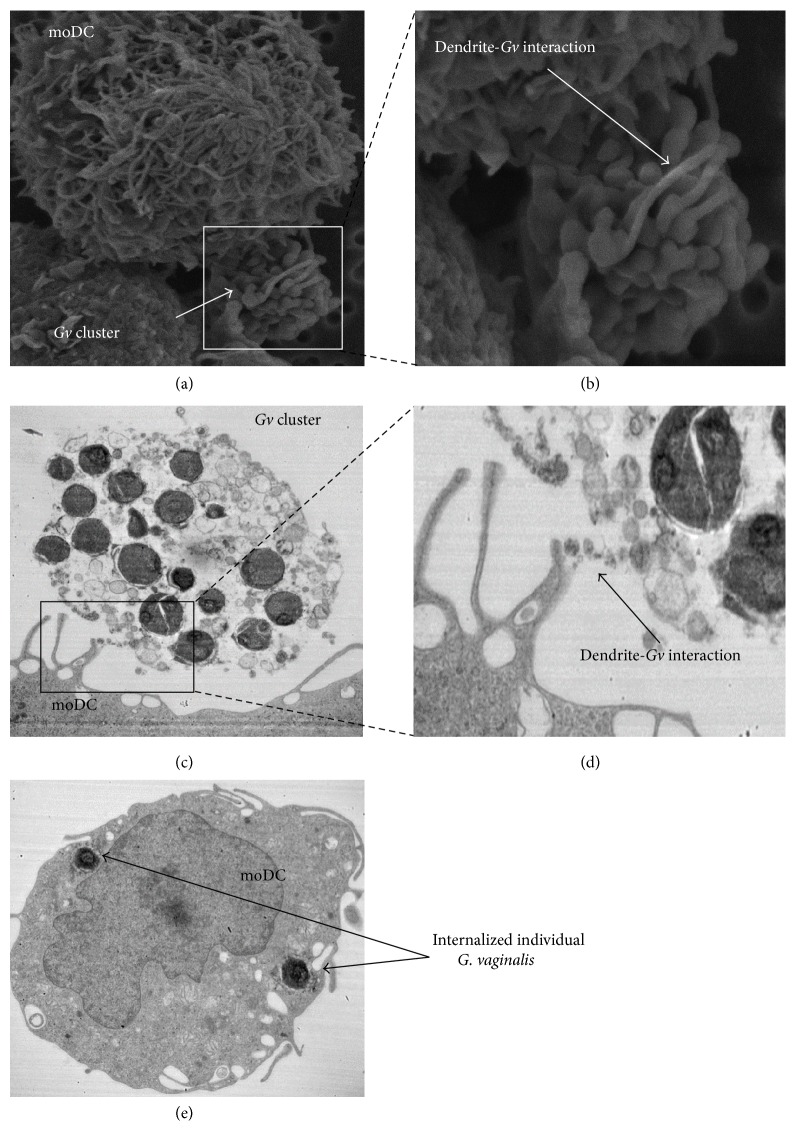
Pictures of DCs after exposure to* G. vaginalis*. (a) SEM (×5500), after a 1 h exposure, DC dendrites surrounding clusters of* G. vaginalis.* (b) Zoom (×13000) on the contact zone between DCs and bacteria. (c) TEM (×12000), after a 3 h exposure, a cluster of* G. vaginalis* in contact with DC dendrites. (d) Enlargement of DC dendrite-*G. vaginalis* interaction zone. (e) TEM cutting, internalized* G. vaginalis *in one DC. moDC: monocyte-derived dendritic cell,* Gv: Gardnerella vaginalis*.

*In vivo*, the first step of the immune response in the vaginal mucosa corresponds to the interactions between bacteria and immature DCs, which lead to the internalization of bacteria. To mimic this initial phenomenon* in vitro,* we put in contact DCs with* G. vaginalis* during 1 to 3 h and took SEM and TEM pictures. SEM produced 3D images of cell and bacteria surfaces after 1 h of contact (Figures 1(a) and 1(b)).* G. vaginalis* bacteria were rarely found on isolated cell forms (in contrast with what we observed with* Lactobacillus, *data not shown) and were in general organized in clusters (Figure 1(a)). However, DC dendrites interacted with this cluster of bacteria and sometimes surrounded it (Figure 1(b)). TEM realized at 3 h, to allow the cells to have time to internalize bacteria, confirmed interactions of DC dendrites with clusters of* G. vaginalis* (Figures 1(c) and 1(d)). Figure 1(c) shows additionally that the cluster is composed of bacteria enrobed in an extracellular matrix which may interact with DCs. TEM also shows internalized intracellular* G. vaginalis *bacteria, but only in their isolated form, without intracellular clusters (Figure 1(e)). At a MOI of 0.01 no DCs with internalized* G. vaginalis *were found, very few at a MOI of 1, and only 35% at a MOI of 100 (count on 100 DCs). For each DC with internalized* G. vaginalis*, the number of bacteria ranged from 1 to 9 bacteria per cell. This experiment was performed in parallel with* L. reuteri* at the same concentrations. Internalization of* Lactobacillus*, unlike* G. vaginalis*, could be observed on few cells at a MOI of 0.01, a majority of cells at a MOI of 1, and up to 82% of cells at a MOI of 100 (count on over 160 DCs). The number of* Lactobacillus* internalized in those cells was higher than with that of* G. vaginalis* since it ranged from 6 to 18 bacteria per cell (data not shown). To conclude on this part, we showed that DCs were able to interact with* G. vaginalis* and to internalize it, but less effectively than lactobacilli, probably due to* G. vaginalis* ability to form extracellular clusters.

### 3.2. Flow Cytometric Analysis of Microbial Effects on DC Phenotype

**Figure 2:**
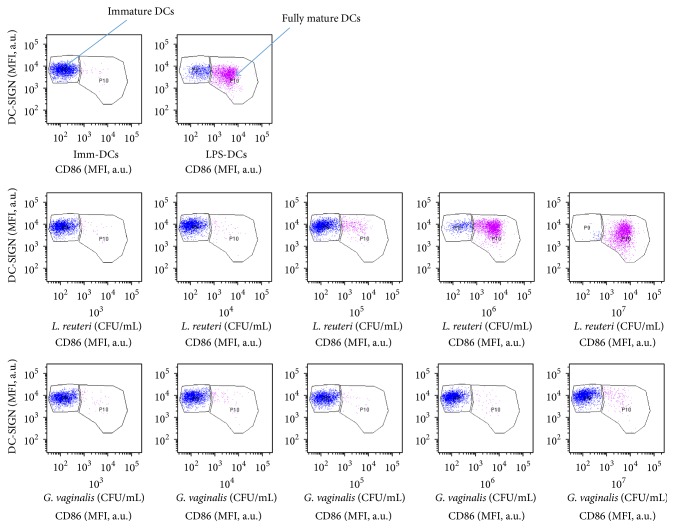
Surface phenotype of human DCs after exposure to a range of* G. vaginalis *or* L. reuteri *concentrations. The dot plots and histograms show MFI values on gated DCs. DC-SIGN/CD86 dot plots gated on human DCs. Data from a representative experiment comparing* G. vaginalis*-induced DC process of maturation with that induced by* L. reuteri*.

**Figure 3:**
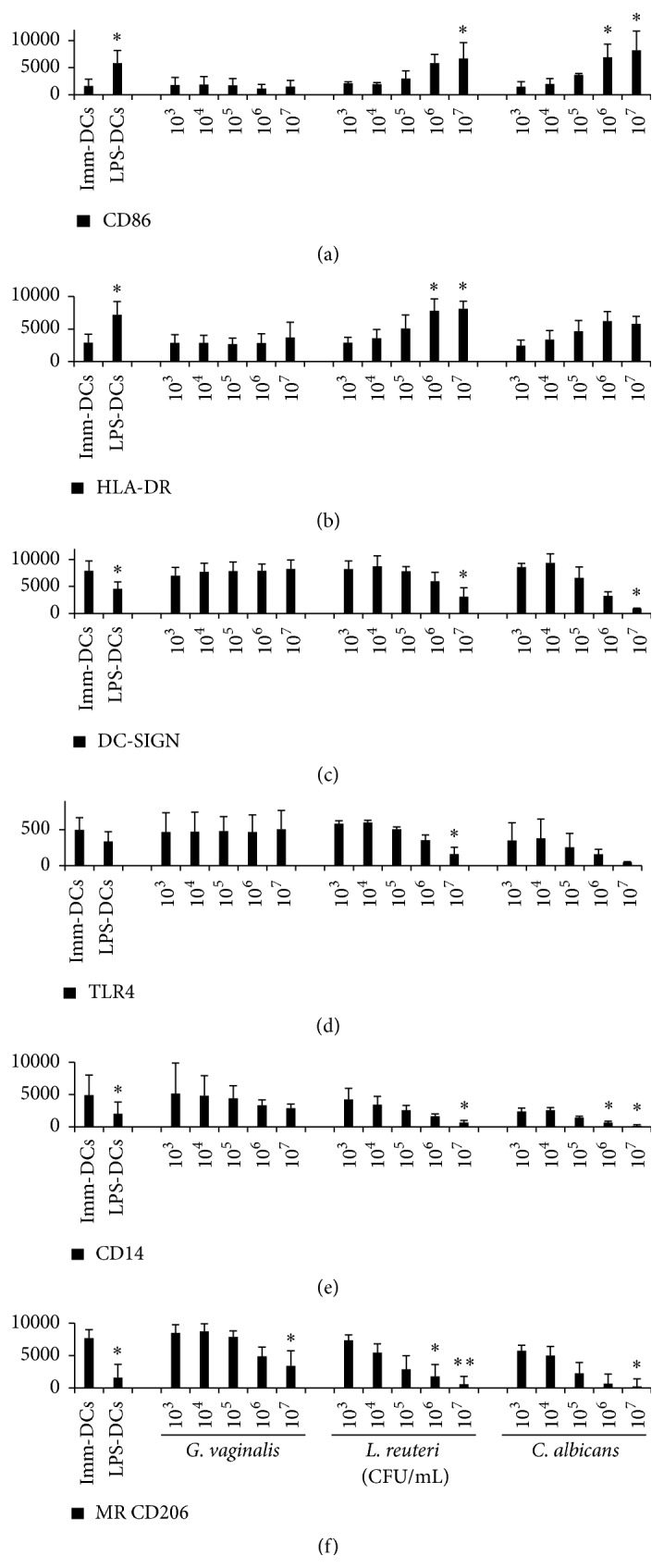
Human DC membrane marker expression after exposure to a range of* G. vaginalis, L. reuteri, *or* C. albicans *concentrations. (a, b) Differential expression of CD86 and HLA-DR, two membrane markers typically increasing during DC maturation. (c, d, e, f) Differential expression of DC-SIGN, TLR4, CD14, and MR CD206, four membrane markers typically decreasing during DC maturation. Data are means (+ SD). For every marker, the isotypic control values were subtracted from the MFI values. Imm-DCs: immature DCs, LPS-DCs: DCs matured by a 48 h exposure to 100 ng/mL* E. coli* LPS;* G. vaginalis*,* L. reuteri*, and* C. albicans*: DCs matured after a 48 h incubation at different concentrations (10^3^ to 10^7^ CFU/mL,* i.e.,* MOI = 0.01 to 100) of* G. vaginalis *(*n* = 5),* L. reuteri* (*n* = 3), and* C. albicans *(*n* = 2). Kruskal-Wallis test with Dunn comparison. ^*∗*^ *p* < 0.05, ^*∗∗*^ *p* < 0.01, as compared to the Imm-DCs.

During the immune response, bacterium internalization can then induce DC activation and maturation, resulting in modifications detectable by cytometry of numerous membrane markers and permitting to distinguish immature DCs from mature ones. In our study, DC activation and maturation were assessed by changes affecting an extensive phenotype of the cell membrane. As expected, immature DCs were characterized by low levels of CD86, HLA-DR, and CD14 expression (the latter was compared to its initial level in monocytes before IL-4 and GM-CSF treatment, data not shown), together with high levels of DC-SIGN and MR expression (Figures 2 and 3). Comparatively, LPS-induced DCs expressed a phenotype characteristic of fully mature DCs with increased levels of CD86 and HLA-DR (Figures 3(a) and 3(b)) associated with decreased levels of DC-SIGN, MR, TLR4, and CD14 (Figures 3(c), 3(d), 3(e), and 3(f)).

Incubation with low or medium doses of* G. vaginalis* (10^3^–10^6^ CFU/mL,* i.e.,* MOI from 0.01 to 10) did not alter immature DC cell surface phenotype, as shown by the absence of significant change in either membrane marker (Figures 2 and 3). At the highest concentration (10^7 ^CFU/mL,* i.e.,* MOI 100),* G. vaginalis* caused a very slight increase in HLA-DR expression (statistically non-significant, Figure 3(b)) combined with moderate decreases in MR and CD14 expressions (both non-significant, Figures 3(e) and 3(f)), which reflect early signs of maturation. However, the lack of increase in CD86 expression and decrease in DC-SIGN and TLR4 expressions indicated that this maturation process was incomplete.

Taken together, our overall cytometric results indicate that* G. vaginalis *induced no maturation of DCs or an incomplete DC maturation process at high doses.

Unlike* G. vaginalis*,* L. reuteri *and* C. albicans *induced a clear dose-dependent higher expression of CD86 and HLA-DR upon maturation of DCs (Figures 3(a) and 3(b)). Combined with decreased expressions of DC-SIGN, MR, CD14, and TLR4 (Figures 3(c), 3(d), 3(e), and 3(f)), these results show that the last two microorganisms induced fully mature DCs, similarly to LPS extract.

### 3.3. Flow Cytometric Analysis of* G. vaginalis* Effects on DC Mortality

**Figure 4:**
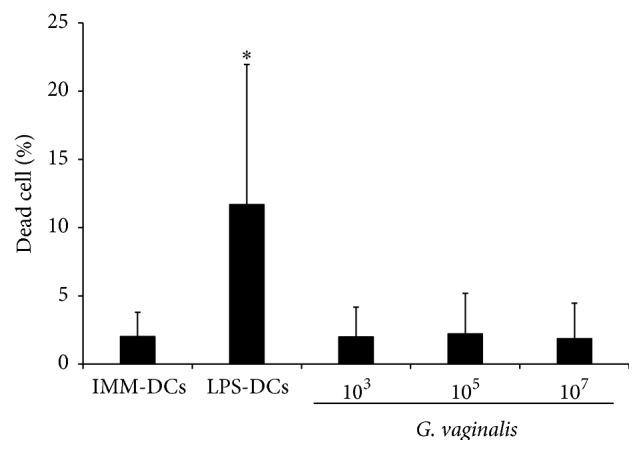
DC viability after 48 h of culture with* G. vaginalis*. Histogram shows the dead cell percentage, calculated from SSC/FSC diagram and live/dead marker. LPS induced a significant increase in DC mortality compared to immature DCs alone in the medium. Low and high concentrations of* G. vaginalis *did not induce any increase in DC mortality, even at a MOI of 100. Data are means (+ SD) from 6 individual experiments. Imm-DCs: immature DCs, LPS-DCs: DCs matured by a 48 h exposure to 100 ng/mL* E. coli* LPS;* G. vaginalis. *DCs matured after a 48 h incubation at different concentrations (10^3^ to 10^7^ CFU/mL,* i.e.,* MOI = 0.01 to 100) of* G. vaginalis.* Friedman's test with Nemenyi's comparison ^*∗*^ *p* < 0.05.

Given the very low level of maturation of DC by* G. vaginalis* observed in our experiments, we hypothesized that this strain would not induce DC cytotoxicity. To test this assertion, a viability marker was added during flow cytometric experiments. Immature DCs alone had almost no mortality. In comparison, exposure of DCs to LPS extract induced a slight, but significant, increase in mortality (Figure 4). Three concentrations of* G. vaginalis* were tested to analyze their impact on DC survival. Results showed that, from 10^3^ to 10^7^ CFU/mL (MOI 0.01 to 100),* G. vaginalis *induced no increase in dead DCs.

### 3.4. Cytokine Secretion by Microbial-Matured DCs

**Figure 5:**
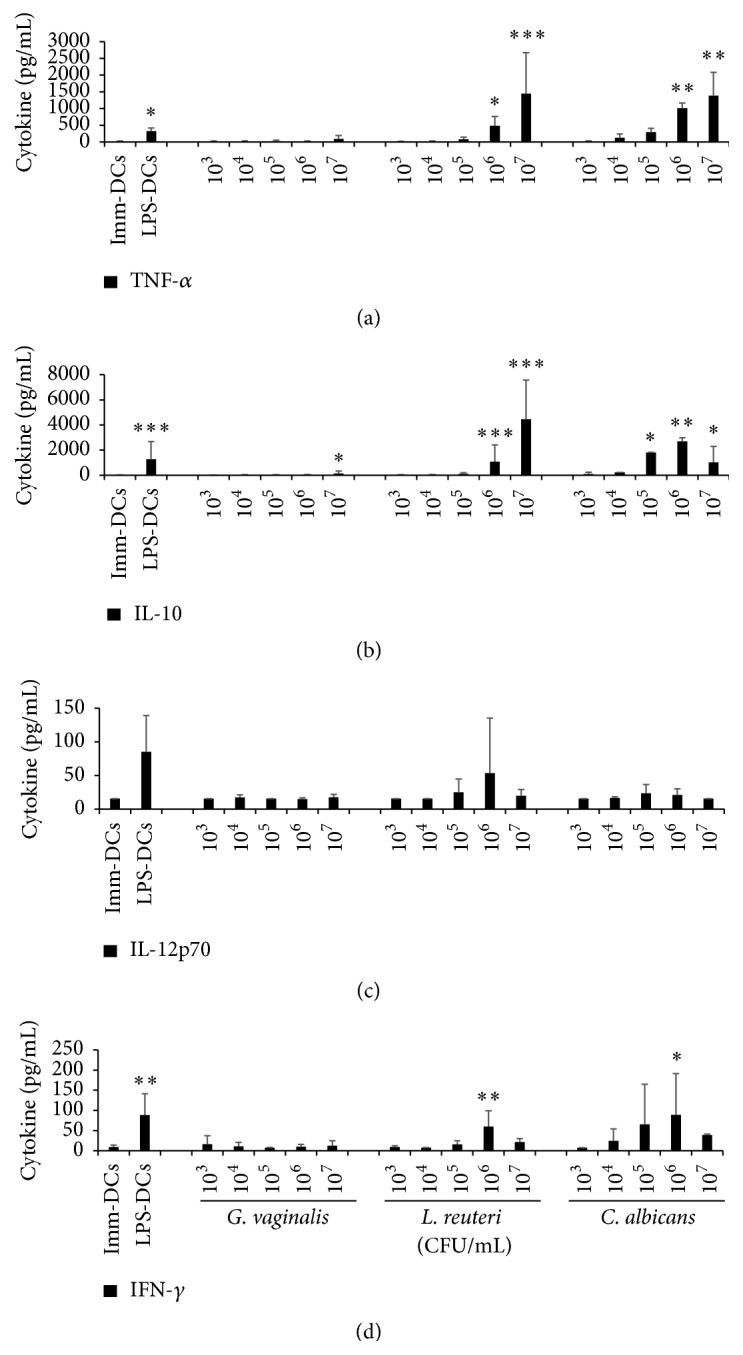
Cytokine production by human DCs exposed to a range of* G. vaginalis, L. reuteri, *or* C. albicans* concentrations. (a, b) TNF-*α* and IL-10 cytokine production.* G. vaginalis* induceda slight increase in IL-10 secretion at high doses but no significant production of TNF-*α*. (c, d) IL-12p70 and IFN-*γ* cytokine production.* G. vaginalis* induced no significant production of the 2 cytokines, even at the highest bacteria concentrations. Data are means (+ SD) of measurements from 6 independent experiments, except for* C. albicans* (3 experiments). Imm-DCs: immature DCs; LPS-DCs: DCs matured by a 48 h exposure to 100 ng/mL* E. coli* LPS;* G. vaginalis*,* L. reuteri*, and* C. albicans*: DCs matured after a 48 h incubation at different concentrations (10^3^ to 10^7^ CFU/mL,* i.e.,* MOI = 0.01 to 100) of* G. vaginalis*,* L. reuteri,* and* C. albicans*, respectively. Friedman's test with Nemenyi's comparison ^*∗*^ *p* < 0.05, ^*∗∗*^ *p* < 0.01, and ^*∗∗∗*^ *p* < 0.001 as compared to the Imm-DCs.

*In vivo,* mature DCs migrate to secondary lymphoid organs and present the antigen to T cells. During this interaction, DCs deliver three signals, notably a cytokinic signal polarizing the differentiation of lymphocytes into several subpopulations. To decipher the preferential pathway of T cell polarization induced by* G. vaginalis,* four cytokines assumed to be of particular interest were selected for ELISA measurements. Compared to untreated immature DCs, DCs incubated with* G. vaginalis* did not significantly increase the production of TNF-*α* or IL-10, except at the upper dose of 10^7^ CFU/mL (Figures 5(a) and 5(b)). Additionally, the production of IL-12p70 and IFN-*γ* was not or barely detectable in* G. vaginalis*-treated or immature DCs (Figures 5(c) and 5(d)). Conversely, a strong dose-dependent increase in the production of TNF-*α* and IL-10 was induced by* L. reuteri *and* C. albicans *and, to a lesser extent, of IL-12p70 by* L. reuteri* and of IFN-*γ* by* C. albicans* (Figures 5(a), 5(b), 5(c), and 5(d)). The fold changes determined by comparing the level of cytokines produced by DCs exposed to 10^7^ CFU/mL* G. vaginalis *(*i.e.,* a MOI of 100) to that produced by immature DCs were equal to 8.9 for IL-10, 6.5 for TNF-*α*, and only 1.1 and 1.3 for IL-12p70 and IFN-*γ*, respectively. By comparison, the fold changes induced by the same dose of* L. reuteri *or* C. albicans *ranged from 70 to 307 for IL-10 and TNF-*α* production. Whatever the concentration of* G. vaginalis*, the level of DC-produced cytokines attained only 10 to 28% of that generated by LPS-treated DCs. Overall, these results show that* G. vaginalis *barely induces cytokinic secretion, in accordance with our findings on DC maturation (Figure 3), thus indicating absence, or slight induction, of DC activation by* G. vaginalis*.

### 3.5. Slight Increase in PHA-Stimulated Lymphocyte Proliferation by* G. vaginalis*

**Figure 6:**
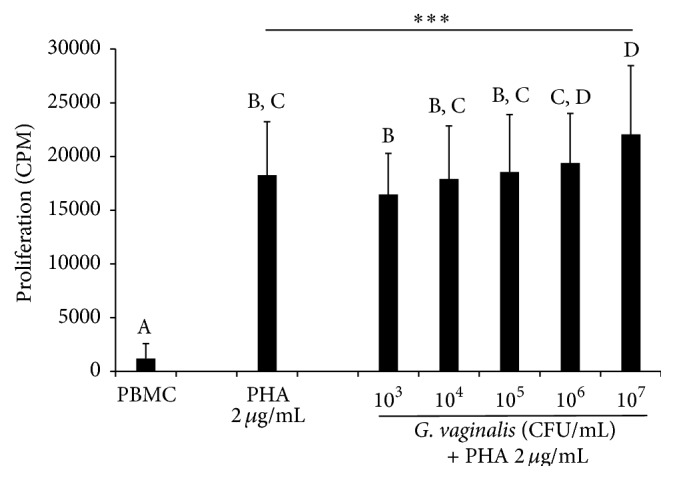
Modulation of PHA-induced lymphocyte proliferation after exposure to a range of* G. vaginalis* concentrations.As expected, 2 *µ*g/mL PHA induced a strong proliferation of the lymphocyte control cells, reaching on average about 20 000 cpm. Compared to the PHA-stimulated control cells, a significant slight increase in lymphocyte proliferation was observed with the* G. vaginalis*-treated cells at a high dose. Data are means (+ SD) of measures from 13 independent experiments. PBMC: PBMCs cultured for 72 h without any effector; PHA control: PBMCs only exposed to PHA (2 *µ*g/mL) for 72 h;* G. vaginalis*: PBMCs exposed for 72 h to PHA (2 *µ*g/mL) in the presence of 10^3^ to 10^7^ CFU/mL of* G. vaginalis*. Two-way ANOVA with* post hoc *Bonferroni test (^*∗∗∗*^ *p* < 0.001). A, B, C, and D: means with different superscript letters are significantly different from each other.

To investigate lymphocyte activation, the next classical stage of the immune response, we carried out functional tests of bacterium-induced modulation of lymphocytic proliferation, using a model of PHA-induced T cell proliferation. Addition of the strain to the medium caused a slight dose-dependent increase in PHA-stimulated lymphocyte proliferation in comparison to lymphocyte control assays without bacteria (Figure 6). The increase in proliferation was only significant at a dose of 10^7^ CFU/mL (*p* < 0.001) and attained an upper average value of 20% compared to that of PHA-stimulated control cells. Lower concentrations of* G. vaginalis *did not cause any significant modulation of PHA-stimulated lymphocyte proliferation. Similar experiments we performed in parallel showed that* L. reuteri *and* C. albicans *did not induce a similar pattern (data not shown).

### 3.6. *G. vaginalis*-Dependent Increase in Cytokine Secretion by PHA-Stimulated Leukocytes

**Figure 7:**
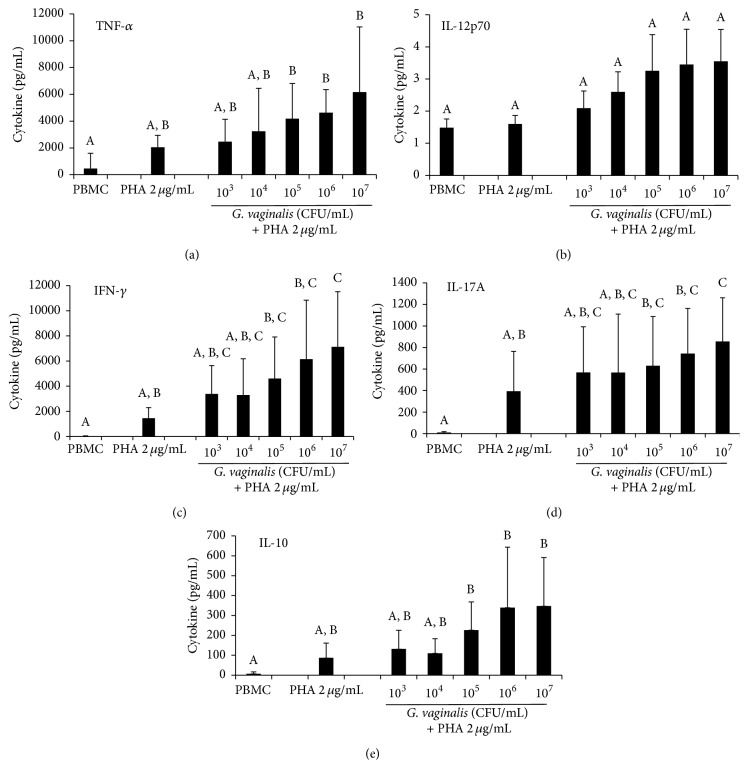
Modulation of PHA-induced cytokine secretion after exposure to a range of* G. vaginalis* concentrations. (a) TNF-*α* secretion (*n* = 6). (b) IL-12p70 secretion (*n* = 8). (c) IFN-*γ* secretion (*n* = 7). (d) IL-17a secretion (*n* = 8). (e) IL-10 secretion (*n* = 8). Compared to the PHA-stimulated control cells, a significant and dose-dependent increase in cytokine secretion was observed for IL-17a and IFN-*γ* with the* G. vaginalis*-treated cells. Other cytokines showed no significant increase in secretion. Data are means (+ SD). PBMC: PBMCs cultured for 72 h without any effector; PHA control: PBMCs only exposed to PHA (2 *µ*g/mL) for 72 h;* G. vaginalis*: PBMCs exposed for 72 h to PHA (2 *µ*g/mL) in the presence of 10^3^ to 10^7^ CFU/mL of* G. vaginalis*. Friedman's test with Nemenyi's comparison (*p* < 0.05). A, B, and C: means with different superscript letters are significantly different from each other.

To depict the type of immune response involved during the* G. vaginalis*-dependent increase in PHA-stimulated lymphocyte proliferation, secretion of cytokines by the four main subpopulations of T cells (IFN-*γ*, IL-4, IL-17A, and IL-10 corresponding to Th1, Th2, Th17, or Treg, resp.) or by APCs (IL-12p70 and TNF-*α*) was measured in extracellular media of lymphocyte proliferation assays. PHA (2 *μ*g/mL) alone induced a strong secretion of IFN-*γ*, IL-17A, IL-10, IL-12p70, and TNF-*α* from PBMCs (Figure 7). A clear dose-dependent increase in IFN-*γ* and IL-17A production was observed in PHA-stimulated PBMCs exposed to varying concentrations of* G. vaginalis* (Figures 7(c) and 7(d)). As shown previously for PHA-stimulated proliferation, only the highest dose of* G. vaginalis* tested induced a significant augmentation in the production of these two cytokines, compared to the PHA control without bacteria. For TNF-*α*, IL-12p70, and IL-10, the increases were non-significant, even at high doses (Figures 7(a), 7(b), and 7(e)). Compared to the control conditions without bacteria, exposure to 10^7^ CFU/mL* G. vaginalis* induced fold increases of 4.9, 4.0, 3.0, 2.2, and 2.2 for IFN-*γ*, IL-10, TNF-*α*, IL-17A, and IL-12p70 production, respectively. Contrastingly, the secretion level of IL-4 was barely measurable or undetectable at any dose of* G. vaginalis *(not shown). Furthermore, at their highest concentration,* L. reuteri *and* C. albicans* caused an increase in cytokine secretion that, depending on the cytokine, was 4- to 5-fold higher than that caused by* G. vaginalis *(data not shown). Overall, these data show that* G. vaginalis *can induce a slight dose-dependent secretion of cytokines on both the inflammatory (IFN-*γ*, TNF-*α*, IL-17A, and IL-12p70) and anti-inflammatory (IL-10) sides.

## 4. Discussion

DCs, the main sentinels of the immune system, are abundant in the human vaginal mucosa, both in the epithelium and in the lamina propria [21]. In this mucosal area, DCs can interact with luminal microorganisms, either indirectly* via* epithelial transport mechanisms or directly* via* dendrites extended across epithelial cells that take up bacteria from the vaginal lumen [31, 32]. As BV is characterized by an imbalance of the normal H_2_O_2_-producing* Lactobacillus *flora toward a polymorphic anaerobic flora with predominant* G. vaginalis*, it is likely that the PRRs of vaginal DCs are subsequently affected by this alteration. To gain insight into the pathophysiological role of* G. vaginalis* in BV, we decided to characterize the interactions between this bacterium and DCs.

We first carried out an electron microscope study of bacteria-DC interactions, the first stage in the immune response, to determine whether* G. vaginalis *can internalize DCs. This was confirmed by using TEM, but we observed that the bacteria were very sparsely represented in free form in culture medium and rather formed clusters coated in an extracellular matrix. As compared to a* Lactobacillus *species which remained in free form,* G. vaginalis* was internalized by DCs much less efficiently. It can reasonably be assumed that this difference is related to the propensity of* G. vaginalis* to form clusters. In addition,* G. vaginalis* forms,* in vivo*, biofilms in the vagina, a process that is involved in BV pathogenesis [33]. These clusters might be the beginning of biofilm formation. Biofilms reduce the host-immune response by decreasing bacteria internalization owing to their large size and to the fact that their extracellular matrix can prevent antigen recognition by APCs [34]. This conformation might thus allow bacteria like* G. vaginalis* to be less internalized by DCs comparatively to strains like* Lactobacillus* that are unable to form biofilms.

We then studied the impact of internalization of* G. vaginalis* on DC maturation status. The maturation state of DCs is a critical determinant of the balance between their tolerogenic and immunogenic abilities [35]. Immature or semi-mature DCs generally promote tolerogenic responses whereas mature DCs promote immunogenic responses [36]. Flow cytometry analysis showed that, whatever bacterial concentrations,* G. vaginalis *elicited minimal changes in the DC membrane phenotype, thus inducing a very incomplete maturation of human DCs. Concurrently, cytokine production remained very low compared to that from LPS-induced fully mature DCs or* L. reuteri- *or* C. albicans-*matured DCs, even at high doses of microorganisms. Taken together, our cytometric and cytokinic data characterize a non-inflammatory DC response at low* G. vaginalis* doses and a very slight pro-inflammatory DC response at the highest concentration.* G. vaginalis* concentrations interacting* in vivo *with mucosal DCs in the human genital tract have not been widely assessed. However, in women with BV, the number of bacteria present can be equal or superior to 10^8^ per mL of vaginal fluid, including about 10^7^ CFU/mL of* G. vaginalis* [37, 38]. Although unknown, the actual number of bacteria in direct contact with vaginal DCs within the mucosa is certainly much lower than this number. Thus, the concentrations of* G. vaginalis *interacting with vaginal DCs in the mucosa of women with BV probably correspond to the low or intermediate MOIs used in our model and causing no or very little DC maturation. The results obtained with* G. vaginalis* were compared to those obtained with two other microorganisms potentially present in the vaginal mucosa, a commensal bacterium (*L. reuteri*) and a pathogenic yeast responsible for mycotic vaginitis (*C. albicans*). Each induced a clear-cut maturation of the DCs, similar to that we previously observed with other pathogens and probiotic strains [39, 40] but in strong contrast to the* G. vaginalis* DC response. In our model,* G. vaginalis *did not induce DC mortality, unlike the two other microorganisms (data not shown). Overall, our findings show that* G. vaginalis* is slightly pro-inflammatory, but less than the* Lactobacillus *strain we used as control.

The effects of* G. vaginalis* on DCs observed in our study were slighter than those reported in other studies, which showed activation and maturation of human moDCs when exposed to the mucosal fluid of women with BV [23, 24]. In these previous studies, DCs were exposed to a mixture of numerous bacterial products secreted by the characteristic polymorphic BV flora and to molecules produced by the mucosal immune system of women with BV. By contrast, in our model, DCs were placed in the presence of* G. vaginalis* alone to find new evidence of its role as a putative BV etiological agent. Thus, the absence of or the very slight* G. vaginalis*-induced DC maturation by* in vitro *direct contact is not inconsistent with a DC maturation induced by substantially secreted products of the overall BV flora, like, for example, the LPS of* Prevotella bivia*. In the vaginal mucosa,* G. vaginalis *could have local specific modulatory effects on immune cells, independently of an overall effect on the maturation of BV flora.

We next performed functional tests of lymphocytic proliferation using a model of PHA-stimulated PBMCs to investigate the activation of lymphocytes, the following stage in the classical immune response. We observed no significant variation in lymphocyte proliferation at all doses of* G. vaginalis* that we used, except the highest one where a significant increase was measured. These findings are consistent with the variations observed in DC status and confirm that* G. vaginalis* induces very few immunologic effects at low doses and a slight pro-inflammatory response at the highest concentrations, which are unlikely to be encountered* in vivo*. This profile of the immunological response to* G. vaginalis *could explain the characteristic lack of external inflammatory signs during BV, in contrast with bacterial or mycotic vaginitis, despite increased pro-inflammatory TLR2 and TLR4 signaling and IL-1*β* secretion reported elsewhere [41–43]. In light of our results, we hypothesize that, depending on its actual amount in contact with the mucosal immune cells,* G. vaginalis* could either go unnoticed or induce low-grade inflammation* in vivo*.

To explore the mechanism of the slight* G. vaginalis*-induced increase in lymphocyte proliferation, a large panel of cytokines was measured in the cell supernatants including molecules secreted by APCs (IL-10, IL-12p70, and TNF-*α*) and/or by T cells (IFN-*γ*, IL-4, IL-17A, and IL-10). We evidenced a similar* G. vaginalis* dose-dependent profile of secretion for all these cytokines, except for IL-4, which remained undetectable. The higher the dose of the pathogen was, the stronger the PHA-stimulation of cytokine secretion was. Thus, this cytokine secretion profile is clearly related to that of the proliferation of PHA-stimulated PBMCs, irrespective of the anti- or pro-inflammatory nature of the cytokines. With regard to the response to* G. vaginalis* of the four main subpopulations of T cells, cytokine secretion suggests a clear dose-dependent induction of Th1 (IFN-*γ*, IL-12p70) and Th17 (IL-17A) and Tregs (IL-10), but not of Th2 (IL-4). Thus, Th1, Th17, and Tregs could be involved in the immune response to high doses of* G. vaginalis*. This topic deserves further investigation in future studies of T cell polarization.

Taken together, our findings show that* G. vaginalis*, by forming clusters and reducing the internalizing ability of DC, induces a slight immunological host response including maturation of DCs, lymphocyte proliferation, and pro-Th1, pro-Th17, and pro-Tregs cytokine production. These immunomodulatory properties are consistent with the atypical clinical profile of BV, which is characterized by a low-grade inflammatory process, as we observed in our* in vitro *model at the highest dose of the bacterium. Our results show the potential immunological effects of the bacteria of the vaginal flora and suggest the existence of a mechanism whereby BV and its associated changes in flora composition could affect host vaginal immunity. Finally, these results lend weight to the putative role of* G. vaginalis *in the pathophysiology of BV and open up broader prospects, in particular for the understanding of the contribution of local immunological alterations to the increased risk of STIs in women with BV.
